# RASSF Signalling and DNA Damage: Monitoring the Integrity of the Genome?

**DOI:** 10.1155/2012/141732

**Published:** 2012-04-11

**Authors:** Simon F. Scrace, Eric O'Neill

**Affiliations:** Department of Oncology, The Gray Institute, University of Oxford, Roosevelt Drive, Oxford OX3 7DQ, UK

## Abstract

The RASSF family of proteins has been extensively studied in terms of their genetics, structure and function. One of the functions that has been increasingly studied is the role of the RASSF proteins in the DNA damage response. Surprisingly, this research, which encompasses both the classical and N-terminal RASSF proteins, has revealed an involvement of the RASSFs in oncogenic pathways as well as the more familiar tumour suppressor pathways usually associated with the RASSF family members. The most studied protein with respect to DNA damage is RASSF1A, which has been shown, not only to be activated by ATM, a major regulator of the DNA damage response, but also to bind to and activate a number of different pathways which all lead to and feedback from the guardian of the genome, p53. In this review we discuss the latest research linking the RASSF proteins to DNA damage signalling and maintenance of genomic integrity and look at how this knowledge is being utilised in the clinic to enhance the effectiveness of traditional cancer therapies such as radiotherapy.

## 1. Introduction

RASSF proteins were originally designated on the basis of sequence homology to domains that associate with Ras-like small GTP-binding proteins. These domains are known as Ras association (RA) domains [RalGDS (Ral guanine nucleotide dissociation stimulator)/AF6 (ALL-1 fusion partner from chromosome 6)] and are distinct from Ras-binding domains (RBD) which bind an alternative set of Ras effectors [1, 2]. Ras belongs to a family of small G-proteins that are ubiquitously expressed and oscillate between an inactive, GDP-bound state, and an active, GTP-bound state, in response to diverse cellular signals. Various GTP-bound Ras-like proteins bind effector proteins to mediate distinct biological responses. There are 150 Ras-like proteins encoded in the human genome which can be grouped by homology or functionality, as being similar to Ras, Rho, Rab, Arf (ADP-ribosylation factor), or Ran. While originally suggested to associate with Ras [3], the RASSF family has a differential affinity for Ras-like GTPases, with NORE1 (RAPL/RASSF5) displaying a much greater affinity for the closely related Ras homolog, Rap1B, than H-Ras itself [4]. The RA domain of RASSF1 associates with K-Ras, rather than H-Ras or N-Ras and is also described to associate with Ran [5, 6]. There are now 10 members in the RASSF family (RASSF1-10) subdivided into two distinct subgroups, the classical RASSF proteins (RASSF1-6) and the N-terminal RASSF proteins (RASSF7-10) based on the location of the RA domain [7]. Little is known about the GTP-binding proteins that may interact with the majority of the RASSF family or how they function but the potential exists for a greater number of signalling connections. In addition to an RA domain, the classical RASSF proteins also have a protein-protein interaction motif known as the SARAH domain that is responsible for scaffolding and regulatory interactions [8]. This domain is a short coiled-coil region and so named due to its location in the extreme C-terminus of genetically linked *Drosophila* proteins; Salvador (hSav1/WW45), dRASSF and Hippo (hMST1/2) (SARAH: SAlvador, RAssf, Hippo) which can form both homo- and heterodimers [9]. The N-terminal RASSFs lack an identifiable SARAH domain, although the SMART database predicts that RASSF7, 8 and 10 contain extensive coiled-coil regions, which can dimerise [10].

RASSF1A and RASSF5A [also known as NORE1A (Novel Ras Effector 1 isoform A)] also contain an N-terminal atypical diacylglycerol/phorbol ester-binding (DAG) domain also known as the protein kinase C conserved region (C1) domain that contains a central zinc finger (Zinc-binding domain) [11]. The Zinc finger in the RASSF family members is denoted “atypical” because it lacks critical residues required for binding of phorbol esters or DNA and therefore probably mediates protein-protein interactions. Indeed, structural analysis indicates that the C1 domain of NORE1A associates with the RA domain to occlude RAS association [12]. As none of the family members have any known enzymatic activity they are thought to be scaffold/adaptor proteins using these binding domains to bring target proteins together to impart their functions.

There are a number of reviews that introduce the RASSF family and the pathways within which they function; however, this paper will focus on the emerging roles of the RASSF family and their effectors in the response to DNA damage. The best described protein in this family with respect to DNA damage is RASSF1 thus the review will concentrate on this protein with particular reference to a recently elucidated signalling network from RASSF1A and the potential clinical significance of targeting this pathway [13].

## 2. RASSF1

It had long been suspected that the 3p21.3 region of the human genome harboured one or more important tumour suppressors because loss of heterozygosity (LOH) was found at this locus in lung, breast, and kidney tumours and genetic instability in this region is the earliest most frequently detected deficiency in lung cancers [14–20]. This 120 kb region contains 8 genes namely *CACNA2D2, PL6, 101F6, NPRL2/G21, ZMYND10/BLU, RASSF1/123F2, FUS1, *and *MYAL2*. However, none of these candidate genes are frequently mutated in cancers [16, 21]. At the same time as these LOH studies, Dammann et al. identified RASSF1 as an interacting partner of the DNA damage repair protein xeroderma pigmentosum complementation group A (XPA) [22]. While the role of RASSF1 in nucleotide excision repair could not be verified, it may yet prove to be significant given the emerging role of RASSF1 in the DNA damage response. The *RASSF1* gene consists of 8 exons spanning a region of about 11 kb. The C-terminal showed high-sequence homology with NORE1, containing an RA domain and thus the gene was named *RASSF1* for Ras association domain family member 1 [22]. Alternative splicing generates 8 isoforms A–H from promoters held within 2 CpG islands. The first CpG island encompasses the promoter regions for RASSF1A, D, E, F, and G. Epigenetic inactivation by DNA methylation at this CpG island is one of the most common events in human cancers (reviewed in [23–25]). This methylation has recently been attributed to HOXB3 driven overexpression of the DNA methyltransferase, *DNMT3B *[26]. RASSF1B, C, and H are generated from a promoter located within the larger 3′ CpG island [27]. This commonly remains unmethylated in cancers and consequently cells retain expression of these isoforms [23]. RASSF1A and RASSF1C are the major transcripts of the *RASSF1 *gene and are expressed ubiquitously in normal tissues [28].

## 3. RASSF1A

Exogenous expression of RASSF1A reduces colony formation in soft agar and reduced tumourigenicity in nude mice [22, 29–31]. Similarly, reexpression of RASSF1A using demethyltransferase inhibitors such as zebularine and 5-aza-2′-deoxycytidine caused significant growth arrest in ovarian cancer cell lines [32]. Reciprocally, RASSF1A knockout mice develop spontaneous tumours, particularly when combined with a knockout of p53, highlighting the significance of RASSF1A in tumour development [33–35]. In addition these RASSF1A^−/−^, p53^−/−^ mice showed high levels of aneuploidy/tetraploidy suggesting an important role for RASSF1A in maintaining genomic integrity. RASSF1A has been shown to have many roles in cell cycle control and microtubule organisation [23, 27], the response to DNA damage is, however, only beginning to be elucidated. It is therefore timely to present these pathways and highlight their importance to the DNA damage response, genomic integrity, and cell survival during cancer development.

## 4. RASSF1 Phosphorylation

The majority of the phosphorylation of RASSF1A has being attributed to the phosphorylation of Serine 202/203. These sites have been demonstrated to be targeted by a number of kinases including, both CDK (Cyclin-Dependent Kinase) and Aurora kinases [36–40]. These phosphorylation events prevent the association of RASSF1A with microtubules during prometaphase. The phosphorylation of RASSF1A on these sites also coordinates the regulation of mitosis by controlling activation of the anaphase-promoting complex/cyclosome (APC/C), and regulation of syntaxin16 to promote cytokinesis [39, 40]. Loss of phosphorylation at these sites leads to defects in mitosis resulting in aneuploidy and genomic instability.

**Figure 1:**
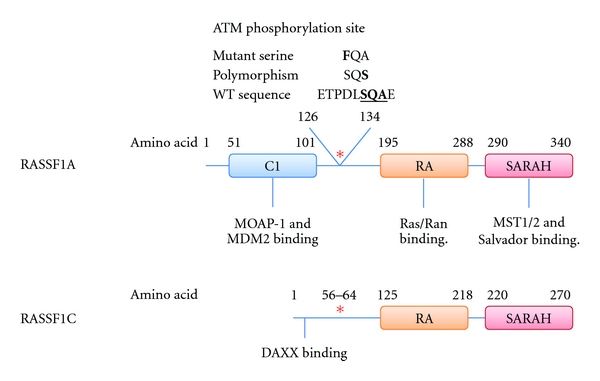
Cartoon depicting the interactions of RASSF1A and RASSF1C. RASSF1A and RASSF1C share a common C-terminal aminoacid sequence, which includes the ATM phosphorylation site (red asterisk), the RA domain, and the SARAH domain but differs at the N-terminal. RASSF1A has a C1 domain which interacts with MOAP-1 and MDM2. RASSF1C lacks the C1 domain but has an alternative DAXX interaction domain. Serine 131 of RASSF1A has been shown to be mutated from serine (S) to phenylalanine (F). An alanine (A) to Serine (S) polymorphism also exists at the 133 site.

In the DNA damage response phosphorylation of RASSF1A serine 131 (S131) is emerging as an important phosphorylation site. The initial kinases that respond to breaks in DNA are the phosphatidyl-inositol 3-kinase like kinases ATM (Ataxia Telangiectasia Mutated), ATR (ATM-and Rad3-Related), and DNA-PK_cs_ (DNA-dependent protein kinase catalytic subunit) [41]. RASSF1A has a consensus site for ATM phosphorylation on serine 131 that is conserved in vertebrates and unique amongst family members and has recently been confirmed as a bone-fide target for ATM [13, 42]. Serine 131 phosphorylation appears important for RASSF1A activation and inactivating mutations of this site have been identified in human cancers [43]. Indeed Shivakumar et al., showed that mutation of the predicted phosphorylation site, S131F, removed the ability to induce cell cycle arrest and block cell proliferation [43]. ATM-dependent phosphorylation at the 131 site is also restricted by S131F and disables the ability of RASSF1A to respond to various DNA-damaging agents [13]. Mutations near the ATM site are hypothesised to function by inactivating ATM phosphorylation. One of these is a nonsynonymous single nucleotide polymorphism (SNP) at p.RASSF1A-A133S (rs2073498), which has significant allele frequencies in human populations (http://hapmap.ncbi.nlm.nih.gov/). The minor allele of the SNP encodes a serine (A133S) and decreases the ability of RASSF1A to become phosphorylated which, like S131F, results in a defective G1 arrest [43]. This suggests that sequence changes to the ATM consensus sequence (aminoacids 125–138) may severely inhibit the function of RASSF1A by disrupting the phosphorylation of S131 and preventing the activation of RASSF1A (Figure 1).

RASSF1A association with Ran directs the formation of a Ran-GTP gradient between the spindle poles and the metaphase plate which is important for the formation of mitotic spindle and for successful completion of mitosis. RASSF1A targets MST1/2 kinase activity towards the RanGEF (GTP exchange factor) RCC1, which inhibits its function and results in elevated Ran-GTP near the metaphase plate. Taken together, these studies indicate that RASSF1A is important for the maintenance of genomic stability by acting as an integrity checkpoint factor. Loss of RASSF1A is likely to weaken the prometaphase checkpoint and increase the potential to create genomic instability and DNA damage leading to cancer development. Indeed, the restriction of RASSF1A activity by modulation of the ATM site may be linked to numerous observations regarding the early onset of tumours in individuals carrying one minor allele of the p.RASSF1A-A133S polymorphism [44, 45]. This has been controversially linked to the exacerbation of a BRCA1/2 genomic instability phenotype; however, the inconsistency may be due to confounding factors other than BRCA2 and may be due to genomic instability via defects in RASSF1A itself  [46]. All this may indicate that DNA damage activation of RASSF1A may provide an extra level of regulatory response, whereby the prometaphase checkpoint senses cells entering into mitosis with DNA damage.

## 5. Regulation by Domain Interaction

As a scaffold, RASSF1A must exert its tumour suppressor function through its interaction domains. The two most important domains in the context of DNA damage are the C1 domain and the SARAH domain. The most significant binding partners identified to interact with the C1 domain are the TNF-R1/TRAIL-R1—Modulator of Apoptosis-1 (MOAP-1) complexes and the MDM2/DAXX/HAUSP/p53 complex [47, 48] (Figure 1). MOAP-1 and RASSF1A are recruited to either TNF-R1 or TRAIL-R1 in response to TNF*α* stimulation. RASSF1A binds MOAP-1 causing an activating conformational change to the structure of MOAP-1. The active structure can bind to the proapoptotic Bcl-2 family member BAX which creates a pore in the outer mitochondrial membrane leading to the release of cytochrome C and induction of caspase-dependent apoptotic signalling pathways [47, 49]. BAX and the associated negative regulator BAK tightly regulate the cell's response to apoptotic signals and are often coordinated with other apoptotic signals such as DNA damage. It is reasonable to assume that RASSF1A-MOAP-1 may be affected by DNA damage but whether this contributes to the regulation of BAX/BAK at the mitochondria remains uncertain.

The response of tumour suppressor p53 to DNA damage results in a variety of outcomes including cell cycle arrest, apoptosis, and senescence, combining to protect the integrity of the genome [50, 51]. In unstressed cells p53 levels are low, being controlled by the RING domain-containing E3 ubiquitin ligase MDM2 (Mouse Double Minute 2) [52, 53]. Induction of DNA damage results in phosphorylation of p53 by the DNA damage checkpoint proteins ATM (on serine 15) and CHK2 (Checkpoint Kinase 2) (on serine 20) [54, 55]. These phosphorylation events combine with an ATM-mediated restriction of MDM2 activity to stabilize p53. Song et al. have recently shown that the C1 domain of RASSF1A can bind and sequester MDM2 in an ATM-dependent manner [48]. They describe a complex consisting of MDM2, DAXX (death-domain-associated protein), and HAUSP1 (a deubiquitinating enzyme). HAUSP1 removes ubiquitin molecules from MDM2 and increases its stability. Upon DNA damage, ATM activates RASSF1A driving its association with MDM2, potentially through phosphorylation at S131. RASSF1A disrupts the MDM2-DAXX-HAUSP1 complex, sequestering MDM2 away from p53, and preventing HAUSP1-regulated deubiquitination of MDM2 promoting its degradation. Release of DAXX from the complex is thought to allow DAXX relocation to the plasma membrane where it can bind the death receptor Fas and activate c-Jun NH_2_-terminal kinase (JNK) [56]. Activated p53 exerts its tumour suppressor function by acting as a transcription factor. It has recently been shown that the *RASSF1* promoter is a target for p53 [57]. Interestingly, p53 appears to downregulate the transcription of RASSF1A hinting at a second mechanism through which p53 can negatively regulate itself in addition to the upregulation of MDM2.

RASSF1A makes two significant interactions through its SARAH domain; the first with mammalian sterile 20-like kinases 1 and 2 (MST1/2) and the second to the scaffold protein Salvador (Figure 1). The RASSF1A interaction with MST1/2 leads to an increase in the local concentration of MST molecules allowing them to undergo transphosphorylation and autoactivation [58]. The interaction further stabilises the MST1/2 kinase activity by preventing dephosphorylation of MST1/2 [59]. MST1/2 were initially cloned from lymphoid cDNA library when looking for human relatives of *Saccharomyces cerevisiae *protein Ste20 and subsequently shown to be activated by a wide variety of cellular stresses [60–63]. Of note is that both *Drosophila* dMST (Hippo) and MST2 are activated in response to DNA damage. In mammals, DNA damage induction of MST2 requires direct binding of RASSF1A- and ATM-mediated phosphorylation of S131 [13, 64, 65]. Interestingly MST1 was shown to be able to activate p53 in response to cisplatin-induced DNA damage by phosphorylating and inactivating Sirt1, a deacetylase that inactivates p53 [66]. Additional substrates of MST kinases that may prove subject to DNA damage are the histones H2B and H2AX, JNK and FOXO transcription factors [67–70]. However, a clear example of signalling through RASSF1A-MST after DNA damage is the recruitment and activation of the large tumour suppressor kinases 1 and 2 (LATS1/2) [71, 72].

Studies on the *Drosophila* homolog of MST1/2, Hippo have discovered that the pathway through Warts (LATS1) is responsible for controlling proliferation and apoptosis and is conserved in both vertebrates and invertebrates. Mutations in pathway member's Hippo (MST1/2), Warts (LATS1/2), Salvador (WW45), or Mats (Mob1 as a tumour suppressor) result in vast tissue overgrowth. The pathway generates a signal to inhibit Yorkie (YAP). Yorkie mutants therefore inevitably show a reduced tissue growth phenotype (reviewed in [73]). Yorkie is a non-DNA binding transcriptional coactivator that binds Scalloped (TEAD1-4) leading to the upregulation of proteins such as Cyclin E and Diap-1 to promote cell division and inhibit apoptosis. In this case Warts phosphorylates Yorkie creating a site for 14-3-3 binding. This sequesters Yorkie in the cytoplasm inhibiting its oncogenic activity [74]. In mammals, in the presence of RASSF1A and a DNA damage signal, LATS1 phosphorylation of YAP maintains a pool of YAP in the nucleus which switches binding partner from the antiapoptotic, YAP-TEAD complex to a proapoptotic YAP-p73 complex [75]. The interaction between YAP1 and p73 stabilises p73 by preventing its nuclear export and subsequent degradation [76–78]. YAP1 functions as a coactivator of p73 and this complex upregulates p73 responsive genes such as the proapoptotic BH3 only Bcl-2 family member, PUMA [79, 80]. This idea is in agreement with the finding that both LATS1 and LATS2 mediate apoptosis through p53. In certain cases LATS2-mediated apoptosis is p53 independent, potentially indicating a switch to YAP1 and p73 [13, 81, 82].

LATS2 has been shown to activate p53 both directly, by binding to and inhibiting MDM2 and indirectly by driving the nuclear accumulation of ASPP1 (apoptosis-stimulating protein of p53) [83, 84]. Interestingly, cytoplasmic ASPP1 appears to behave in an opposite manner and inactivates the ability of LATS1 to interact with YAP1 [85]. As RASSF1A activates LATS1/2 in response to DNA damage this could potentially drive ASPP1 activation of p53 and contribute to the overall p53 response. Interestingly the *Drosophila *ASPP protein (dASPP) has also been shown to interact with dRASSF8 to regulate C-terminal Src kinase (dCsk) and adherens junctions [86], a site key to the regulation of the core hippo pathway [87].

LATS2 has been implicated in the G1 tetraploidy checkpoint, a process that is thought to be driven by LATS2 activation by ATR and leads to direct stabilisation of p53 [83, 88]. Active p53 then creates a positive feedback loop with LATS2 by upregulating its activity further [88]. In response to UV radiation CHK1 activation by ATR has been shown to activate LATS2 [89].

Although not addressed in a RASSF1A-dependent manner, YAP forms an additional DNA damage promoted complex with the transcription factor early growth response 1 (EGR1) [90]. The interaction promotes enhanced transcriptional activity of EGR1 for the Bcl-2-associated X (BAX) promoter. Thus YAP can act as an oncogene and a tumour suppressor in a RASSF1A-context-dependent manner.

In *Drosophila* dRASSF and Salvador are known to compete for MST binding. Here Salvador acts as an adaptor to bring Hippo and Warts together to activate the hippo pathway, which is antagonised by dRASSF [91]. In mammals, however, RASSF1A can bind both MST1/2 and Salvador at the same time using different regions with the SARAH domain. Using an L308P mutant of RASSF1A that cannot bind MST but remains bound to Salvador, Donninger et al. have shown that the RASSF1A Salvador interaction can activate p73 in an MST-independent manner [92].

## 6. RASSF1C

RASSF1C is the second ubiquitously expressed isoform of the *RASSF1* gene. Like RASSF1A, RASSF1C contains the ATM consensus sequence (Figure 1). This site, at Serine 61, has not yet been confirmed but the sequence is identical between RASSF1A and RASSF1C at this site so it is plausible to suggest that RASSF1C is also phosphorylated and activated by ATM. Indeed, the Serine 61 to phenylalanine (S61F) mutant of RASSF1C was unable to block the genomic destabilising effects of Ras which can be ablated by overexpression of wildtype RASSF1C in the embryonic kidney cell line 293T and human lung tumour cell line NCI-H1299 [93]. This suggests that DNA damage activation of RASSF1C may require phosphorylation of Serine 61 (RASSF1A-131) site. Further to this, RASSF1C has recently been implicated in a DNA damage response pathway involving DAXX (which also binds to RASSF1A) and JNK [94] (Figure 1). In unstressed conditions RASSF1C is shown to be in a complex with DAXX in the nucleus, recently resolved by NMR [95]. Upon ultraviolet radiation or MMS-induced DNA damage this interaction is lost allowing RASSF1C to move to the cytoplasm where it aids the activation of SAPK/JNK signalling [94]. DAXX, however, remains in the nucleus concentrating at PML bodies. The signal that leads to release of RASSF1C from DAXX is unknown; however, it would be interesting to see if the signal relies upon the ATM phosphorylation site. Conversely, another study has identified that RASSF1C, far from being activated by DNA damage, is targeted for degradation under stress conditions. Exposure to UV radiation or treatment of cells with doxorubicin leads to RASSF1C phosphorylation by GSK3*β* creating a phosphodegron at S19/23 which is bound to by SCF^*β*-TrCP^ targeting RASSF1C for degradation [96]. This GSK3*β*-dependent degradation was shown to be inhibited by the PI3-K/AKT pathway. Since AKT activity can lead to RASSF1C upregulation it suggests that RASSF1C could function as an oncogene. This is in keeping with several recent reports showing that RASSF1C increased cell proliferation in lung cancer cells and migration in breast cancer cell lines [97, 98].

## 7. Therapeutic Implications of RASSF1A Loss

One of the most common and widespread events to occur during cancer development is the loss of RASSF1A expression. This loss is due to methylation of the upstream CpG islands in the *RASSF1* gene [22, 29]. The frequency of epigenetically driven loss of RASSF1A correlates well with the increasing grade of the tumour. Methylation has been reported in over 37 tumour types (comprehensively reviewed in [24, 99]) and is thought to be an early event in breast and thyroid tumourigenesis, childhood neoplasia, and endometrial carcinogenesis [27].

RASSF1A methylation correlates with a decreased responsiveness to DNA-damaging therapies [100–102]. The DNA methyltransferase (DNMT) inhibitor zebularine has been used to effectively reexpress RASSF1A and show an increase in cancer cell sensitivity to radiation-induced damage *in vitro *and *in vivo* [101] as well as to cisplatin [32]. Dote et al. showed that 48 h treatment with zebularine, which corresponded to the maximum reexpression of RASSF1A increased the radiosensitivity of PaCa, DU145, and U251 cancer cell lines by 1.5 times and caused an increased tumour delay in U251 xenograph models in mice [101]. A 48 h treatment with zebularine also increased cancer cell sensitivity to DNA damage and a 16-fold reduction in IC_50_ of cisplatin in resistant ovarian cancer cell lines [32]. Sensitivity of testicular germ cell tumours to cisplatin could also be enhanced by another DNMT inhibitor that is in clinical trials, 5-aza-2′-deoxycytidine [103]. Interestingly, they noted that effectiveness of the 5-aza-2′-deoxycytidine treatment was dependent on the level of DNMT3B levels. The higher the DNMT3B level the greater the effect. The most significant target gene for DNMT3B was shown to be RASSF1A (as mentioned above) and thus it can be extrapolated that the increase in sensitivity to cisplatin is due to the reexpression of RASSF1A. Reexpression of RASSF1A using 5-aza-2′-deoxycytidine or reintroduction of RASSF1A into the hepatocellular carcinoma cell line, SMMC-7721, was also shown to increase sensitivity to chemotherapeutics such as fluorouracil, mitomycin, and cisplatin [104]. Together these results support a clinically relevant role for RASSF1A in the DNA damage response that is backed up by phase I and II clinical trials in myelodysplasia and leukaemia patients where 5-aza-2′-deoxycytidine has shown efficacy both alone and in combination with the histone deacetylase (HDAC) inhibitor valproic acid [105, 106]. Therapeutic failure upon RASSF1A loss can also be counteracted by targeting the downstream DNA damage responsive signalling pathway. Direct activation of BAX via the BH3 mimetic ABT-737 has recently put forward as a potential treatment for RASSF1A methylated medulloblastoma [107]. The role of RASSF1A in checkpoint activation and maintenance of genomic integrity is highlighted in a study by Zhang et al. which showed a significant increase in DNA damage caused by aflatoxin B_1_ in tumour tissues where RASSF1A has been lost due to DNA methylation [108].

## 8. Other RASSFs and DNA Damage

This paper has concentrated primarily upon the role of RASSF1 in DNA damage; however, it is worth noting that other RASSF proteins have also been linked to DNA damage pathways. The RASSF2 gene resides on chromosome 20. The gene can be spliced into two very similar proteins RASSF2A and RASSF2C both of which contain the RA domain and the SARAH domain. They show 28% identity to RASSF1A and like RASSF1A, the promoter has been shown to be inactivated by hypermethylation in primary tumours [109–114]. RASSF2 has been reported to be upregulated in lymphocytes from individuals exposed to ionising radiation [115]. RASSF2 has also been shown to associate with, and is phosphorylated by, MST2 leading to stabilisation of MST2 and the generation of proapoptotic signals [116].

The *RASSF6* gene is located on chromosome 4. While the expression of RASSF6 is lost in cancer, *in silico* analysis did not find any CpG islands located near the promoter; therefore, it is assumed that this loss is not due to DNA methylation [117, 118]. RASSF6 is known to activate apoptosis in both caspase-dependent and -independent mechanisms in response to TNF*α*; however, it is unknown whether it is also activated by DNA damage signals [118]. RASSF6 contains both the RA domain and SARAH domain and like RASSF1A it has been shown to bind to MOAP-1 [117], which could be responsible for its induction of apoptosis in response to TNF*α*. Unlike other family members, RASSF6 contains a number of ATM consensus sites (SQ/TQ) upstream of the RA domain; however it is not clear if these are functional.

RASSF family members efficiently form heterodimers [119]. This provides a potential mechanism through which additional RASSF proteins could be involved in DNA damage signalling. A heterodimer between RASSF1A and RASSF5A has been suggested to be important for the interaction of RASSF1A with Ras [120]. Given that each of the RASSF proteins above is thought to impart its tumour suppressor function through the MST kinases we could propose that heterodimeric interactions between RASSF family members may be important for their DNA damage-induced apoptotic signalling.

RASSF7 is the best studied N-terminal RASSF protein and the first to be shown to be linked to the DNA damage response. Located on chromosome 11 close to the H-Ras gene (*HRAS1*), it forms part of a microsatellite that is associated with increased cancer risk [121–123]. Unlike the majority of the RASSF family members that are silenced in cancer, RASSF7 has been shown to be upregulated in a number of cancers including pancreatic, endometrial, and ovarian [124–128]. The upregulation of RASSF7 in cancers suggests an oncogenic function, the mechanism of which has only just started to be explored. RASSF7, in concert with N-Ras, is thought to suppress the activation of JNK in response to low doses of UV radiation by binding and inhibiting MKK7, preventing its interaction with JNK. At higher doses of UV, RASSF7, like RASSF1C, is targeted for degradation through an ubiquitin-dependent mechanism. This frees MKK7 to activate a stress response through JNK [129].

## 9. Conclusion

**Figure 2:**
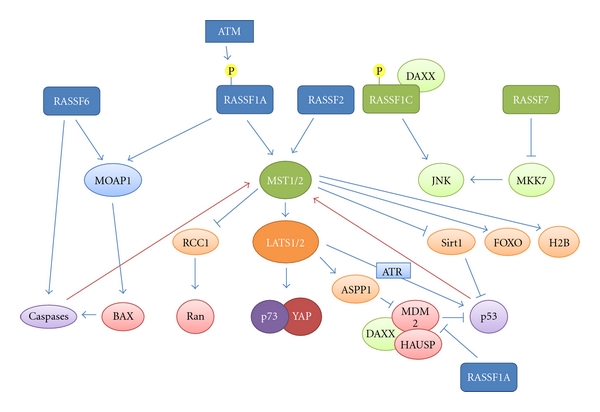
Cartoon depicting DNA damage activated pathways downstream of RASSF family members. RASSF family members, activated by DNA damage, signal through various intermediates (primary interaction: light blue and green [involving RASSF1C or RASSF7]; secondary: orange and tertiary: red) to activate p53, p73, and caspases (purple) to control apoptosis, genome stability, and senescence. Feedback loops exist from caspases and p53 that further activate the pathways and amplify the signal. RASSF1A can also directly sequester MDM2 leading to p53 activation. RASSF1C can transfer DNA damage signals from the nucleus to the cytoplasm by activating JNK signalling. RASSF7 acts as an oncogene inhibiting the activation of JNK.

Ras-association domain containing family members are important tumour suppressors involved in linking cellular stresses to cell cycle arrest and apoptosis (Figure 2). RASSF1A is an adaptor protein with three major interaction domains through which it imparts its functions. Each of these domains is involved in binding different effector proteins in response to DNA damage. The C1 domain binds MDM2 to stabilise p53 and the RA and SARAH domains are required to activate the mammalian Hippo pathway. The mammalian homolog of Hippo, MST1/2, can activate apoptosis in response to cellular stresses either directly, in the case of FOXO1 and histone H2B or via LATS1/2. RASSF2, RASSF5, and RASSF6 which share the RA and SARAH domains with RASSF1A have also been shown to active MST1/2 to induce apoptosis as well as being able to induce apoptosis independently of the Hippo pathway. LATS1 and 2 have been implicated in apoptosis by stabilising both p53, either directly through an interaction with MDM2 or indirectly via ASPP1 and stabilising p73 via YAP, in response to DNA damage. RASSF1C has been shown to be released from DAXX and p53 upon DNA damage where it can go and transmit the damage signal from the nucleus to the cytoplasm by activating JNK signalling. Each of these proteins appears to act both upstream and downstream of the “guardian of the genome” p53 to create a network which feeds back upon itself to enhance the DNA damage signaling within the cell. Greater than 50% of human tumours has either lost or mutated p53. Disruption of these networks will inactivate p53 and may contribute to tumourigenesis in a number of the cases where wild-type p53 is retained. Although not correlated with p53 loss or mutation, RASSF proteins are epigenetically lost in human cancers by DNA methylation. It has been shown that, as with p53, loss of *RASSF1* expression is associated with more aggressive tumours and increased resistance to radiation-induced DNA damage and platinum-based drugs. DMNT inhibitors such as zebularine have been shown to reexpress RASSF1A and increase the radiosensitivity of these cancers suggesting that reexpression of RASSF1A and other silenced RASSFs maybe a path through which chemoradioresistant tumours can be combated.
